# Impact of genetic variants on B-cell development and function in systemic lupus erythematosus

**DOI:** 10.1186/ar4624

**Published:** 2014-09-18

**Authors:** Karen Cerosaletti, Tania Habib, Richard James, Janice Chen, Melissa Pickett, Andrew Funk, Shan Wei, Jane H Buckner

**Affiliations:** 1 Benaroya Research Institute at Virginia Mason, Seattle, WA, USA; 2 Seattle Children's Research Institute, Seattle, WA, USA

## Background

Systemic lupus erythematosus (SLE) is an autoimmune disorder characterized by the progressive loss of tolerance to nuclear antigens and the production of pathogenic autoantibodies. Genetic polymorphisms in genes involved in B-cell signaling, *PTPN22, CSK, BLK *and *BANK1*, are associated with susceptibility to SLE.

## Methods

We have utilized a cohort of genotyped healthy subjects to better understand how these genetic variants contribute to the failure of B-cell tolerance seen in SLE. PBMC from these subjects are analyzed using multiparameter flow cytometry to assess the composition of the B-cell compartment and the response of B cells to stimulation via BCR and CD40.

## Results

Our studies in healthy subjects who carry this variant have demonstrated alterations in the composition of the transitional and naïve B-cell pool. This is functionally correlated with the altered BCR response and enhanced survival of these cells in carriers of the risk variant. Three potentially functional *BANK1 *SNPs (including a splice branch point-site variant and two coding variants) are associated with SLE. We have shown that the *BANK1 *risk variants are associated with homeostatic changes in the peripheral B-cell pool that include a significant expansion of the total memory and preswitch memory compartment; and a significant decrease in naïve B cells in subjects homozygous for the *BANK1 *risk alleles. In further studies we have demonstrated that the *BANK1 *splice variant is associated with significantly reduced expression of the Δ2 isoform and that the risk haplotype is further associated with blunted proximal BCR signaling in naïve B cells and enhanced p-AKT in memory B cells. Studies are ongoing to investigate the impact of the *BANK1 *variant on plasma cell differentiation.

## Conclusions

Studies of healthy subjects who carry SLE risk genes demonstrate alterations in B-cell function and fate. These studies can then be extended to subjects with SLE to understand how these genetic variants impact B-cell development and tolerance in the setting of disease.

